# Effect of Bleaching on Surface Roughness of Universal Composite Resins After Chlorhexidine-Induced Staining

**DOI:** 10.3390/dj13070277

**Published:** 2025-06-20

**Authors:** Gözde Aksoy Vaizoğlu

**Affiliations:** Department of Restorative Dentistry, Section of Dentistry, Kyrenia University, North Cyprus 99300, Turkey; gaksoy432@gmail.com

**Keywords:** office bleaching, CHX solution, surface roughness, universal composite resin, 3D profilometer

## Abstract

Objective: The aim of this study was to compare the surface roughness of a conventional composite resin, a one-shade universal composite resin, and a group-shade universal composite resin after bleaching. Methods: A conventional composite resin, Clearfill, and the universal composite resins Omnichroma and Optishade were prepared into discs (2 × 8 mm), with a total of 90 discs. Each group (30) contained three different groups, including a control (n10), coloured with CHX (n10), and after bleaching (n10). The surface roughness of group discs was measured with a 3D profilometer. ANOVA and Kruskal–Wallis Tests were used to analyse data. Analyses were performed in the SPSS programme. Results: Significant Ra values were obtained between subgroups (*p* < 0.05). Comparisons of surface roughness after discolouring were performed with the control group; the highest surface roughness value (*p* > 0.05) was found for Optishade after bleaching compared to the control group. The Optishade composite resin showed the highest initial surface roughness value (*p* > 0.05), and after discolouration followed by bleaching, the Clearfill composite resin showed the highest surface roughness value (*p* > 0.05). Conclusions: Both immersion and bleaching applications cause surface roughness. As a result, it was determined that the composite resin content has a significant effect on the surface roughness in discolouring and bleaching processes.

## 1. Introduction

Patients’ aesthetic expectations regarding composite resin restoration are increasing day by day. Along with this, expectations regarding the development of composite resins are also increasing [1]. An ideal composite resin should not wear, should provide good retention, should provide good aesthetics, and most importantly should not discolour. Anterior composite resin restorations, which are preferred over porcelain or zirconium, should have superior properties [2].

Composite resins can be discoloured due to intrinsic or extrinsic reasons. While intrinsic discolouration is mostly caused by systemic diseases displayed by the patient, many factors can cause extrinsic discolouration. The patient’s eating habits, smoking habits, and toothpaste and mouthwash use are examples of these factors [3]. In particular, patients with poor oral hygiene are often known to have used a mouthwash in their dental history in accordance with their dentist’s recommendations or by self-supply. It is often observed that these patients exceed the recommended duration and frequency of use of mouthwash [4], and it is observed in clinics that this can lead to their teeth becoming discoloured, especially after aesthetic composite resin restoration. Following this discolouration, surface roughness increases and plaque formation, further discolouration, and the need to renew the restoration over time are observed [5]. Surface roughness is still considered a disadvantage of composite resins. Surface roughness, which is minimised with a good finish and polish, may increase with the effect of external factors [6]. In order to maintain clinically appropriate surface smoothness, the average filler size should not exceed 1 µm and the maximum particle size should be limited to 0.5 µm. Other important filler properties that affect surface quality and wear resistance include the composition of the adhesive and the surface hardness after polymerisation. Microfiller composites have superior surface properties than most small-particle hybrid composites. Nevertheless, they display more wear. For these reasons, the current trend is to reduce the particle size of fine-particle hybrids in order to achieve ideal surface properties without loss of physical properties of microfiller composites. It has also been reported that the main factors affecting the surface roughness are resin particle size, type, and density [7]. The particle size of standard hybrid composites, called conventional composite resins, varies, but can reach 1.5 µm or more. Traditional composite resins have been replaced by nanotechnological nanocomposite resins [8]. Nanocomposite resins have advantages such as lower polymerization shrinkage, higher optical properties, higher surface smoothness, and higher colour stability. Universal composite resins can have supranano particle sizes in combination with nanotechnological nanoparticles [9]. Therefore, two composites with different properties were compared in our study.

Chlorhexidine (0.2 CHX) mouthwash is undoubtedly one of the most commonly used mouthwashes. It is the most common type of mouthwash used to destroy bacteria in the oral cavity in people with oral disease or for disease prevention [10]. It is easily available from pharmacies and grocery stores, providing easy access for patients. However, it should be used carefully [11,12]. The discolouration caused by chlorhexidine mouthwashes has been demonstrated by many scientific publications, but further studies on their effect on surface roughness are still required. Meanwhile, their effects on today’s universal composite resins are still being investigated.

Bleaching is a procedure preferred by physicians as the easiest treatment for discolouration. There are many bleaching procedures and materials available. Whitening can be performed in the form of home bleaching and office bleaching. Office bleaching, performed by physicians, is always more advantageous, because it has advantages such as the duration of use, length of the session, controllability, and lack of soft tissue irritation. It is known that whitening applied at home has many disadvantages that may occur after unconscious errors by the patient [13]. The active ingredient in tooth bleaching agents is hydrogen peroxide and, as a strong oxidising agent, it causes the formation of free radicals, reactive oxygen molecules, which break the pigmented molecules into small fragments, allowing them to diffuse to the outer surface of tooth or to be seen in a lighter colour by absorbing less light. Hydrogen peroxide does not cause any significant change in the organic and inorganic content or crystal structure of the tooth, but it oxidises the organic matrix [14,15].

The aim of our study was to investigate the comparative effect of chlorhexidine mouthwash on two types of universal composite resins and one conventional composite resin, as well as to investigate the comparative effect of a hydrogen peroxide agent on coloured composite resins.

## 2. Materials and Methods

This study investigated three different composite resins: a conventional multi-shade hybrid composite resin (Clearfill Majesty Esthetic, Kuraray, Okayama, Japan), a group-shade nanohybrid universal composite resin (Optishade, Tokuyama Dental, Encinitas, CA, USA), and a one-shade supranano spherical universal composite resin (Omnichroma, Tokuyama Dental). Diamond particulate Twist Dia Spiral Wheels (Kuraray, Okayama, Japan) were used to finish and polish the composite resin disc. The immersion solution in this study was chlorhexidine mouthwash (Klorhex, Balgat, Ankara), and the bleaching agent was Bleaching Smile Automix bleaching gel (%35 HP, ph 7.3) (Schütz Dental, Rosbach, Germany).

Composite specimens (8 mm in diameter and 2 mm in thickness, *n =* 90) were prepared using metal moulds. A mylar strip was used under the mould and composite resin was applied in the hole. After applying the composite resin, it was pressed with a glass slide to obtain a flat surface. The specimens were cured for 20 s using a LED light-curing unit (Woodpecker, Led B, Curing Light, Guilin, China) with a light intensity of 1000 mW/cm^2^. The specimens were removed from the mould and assessed visually for any structural defects. Specimens were stored at 37 °C in distilled water for 24 h to complete polymerization.

**Figure 1:**
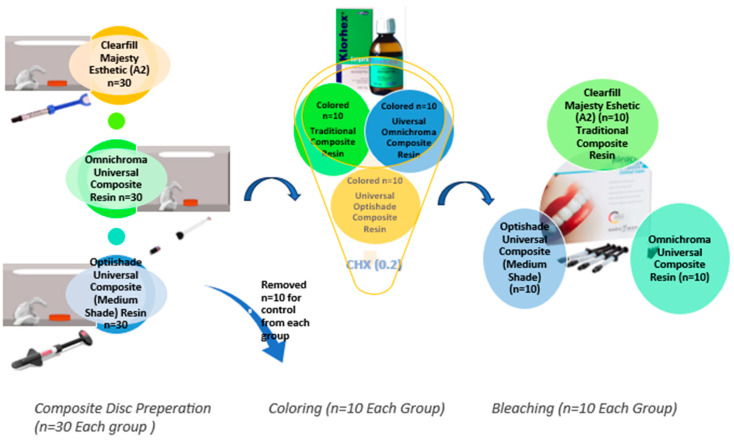
Research schematic.

After polymerization was completed, the specimens were finished and polished for the same amount of time under the same pressure conditions with Twist Dia Spiral Wheels. Each composite resin group was divided into three subgroups (*n =* 30): control, immersed, and bleaching. The control (*n =* 10) group was used for comparison with the results from the other groups. The second (*n =* 10) group was immersed in a 0.2% CHX mouthwash solution for 24 h, which is reported as being the equivalent time to 2 years of daily use for 2 min [16]. The third group (*n =* 10) was bleached after the completion of the immersion time and washed with distilled water according to the manufacturer’s instruction. Figure 1 presents the research schematic of this study.

The surface roughness of specimens was evaluated with a profilometer (Staylus Profilometer, NanoMap, LS, Nanshan, China) for each group. The surface roughness (arithmetic mean roughness–Ra) of the specimens was examined with a profilometer in contact mode. The surface roughness values were obtained using the diamond tip of the profilometer, 2.5 µm in radius, with a cut-off value of 0.25 mm, a speed of 75 µm/s, and a range of 500 µm. This procedure was performed on 3 different sides and the mean Ra values were obtained for each specimen.

### Statistical Analysis

Based the main aim of the research, we planned to investigate the differences between independent groups and to examine their changes over time. Similar studies that could be used in sample size calculation were examined and the sample size calculation that provided the highest number according to the statistical methods to be applied in line with the main aim was taken into consideration. In this study, using the ‘G. Power-3.1.9.2’ programme [17], at a 95% confidence level (α = 0.05), the standardised effect size was taken as 0.25 [18] due to the lack of similar studies, while the minimum sample size was obtained as 10 for each group and time (9 groups and 3 times), with a theoretical power of 0.95.

The assumption of normal deviation was checked using the Shapiro–Wilk test and homogeneity of variance was checked for by means of Levene’s test. ANOVA was used to compare three or more independent groups with a normal frequency distribution and the Kruskal–Wallis test was used when there was no normal frequency distribution. Post hoc Bonferroni and corrected Bonferroni tests were used to reveal the group or groups that made the most difference. Analyses were performed in the IBM SPSS 27 programme.

## 3. Results

The results of Ra measurements according to the composite, CHX, and bleached groups are presented in Table 1, and ANOVA and Kruskal–Wallis tests were used for comparison. In the analyses performed for the Omnichroma, Optishade and Clearfill groups, significant differences were found in the comparison between each subgroup. A significant difference was found in the Omnichroma and Optishade composite resins after immersion compared to the control groups. A significant difference was found in the bleached subgroup of the same groups after discolouring. The Omnichroma and Optishade immersed groups had greater surface roughness than the corresponding bleached groups. In the Clearfill composite resin group, a statistical difference was found between the subgroups. However, compared to the other groups, surface roughness measurements after staining were significantly lower than surface roughness measurements after bleaching.

The distributions of Ra measurements according to the composite and bleaching methods are presented and compared in Table 1. To provide a better understanding of these results, we can analyse this table in three ways.

1. Comparison of surface roughness after discolouring compared to control group: The highest surface roughness was found for Optishade. This was followed by the Clearfill composite resin, while the Omnichroma group showed the lowest surface roughness value.
2. Comparison of the surface roughness after bleaching with the control group: The Optishade composite resin showed the highest surface roughness value. The lowest surface roughness value was found for the Clearfill composite resin.
3. Comparison of surface roughness after discolouration with surface roughness after bleaching: The Clearfill composite resin showed the highest surface roughness value. This was followed by the Omnichroma composite resin and the Optishade composite resin, which displayed the lowest value.

As a consequence, when these results are considered, the composite resin with the highest deterioration in surface roughness after discolouring was found to be Optishade. The Clearfill composite resin ranked second in surface roughness, with a slight difference after discolouring. After bleaching following discolouring, which represents the main focus of this study, the Clearfill composite resin showed the highest surface roughness value, with a high intermediate difference. Intermediate 3D images of the surface roughness are presented in Table 2. As seen in the 3D illustrations, the larger the red area, the more surface roughness is observed.

**Figure 2:**
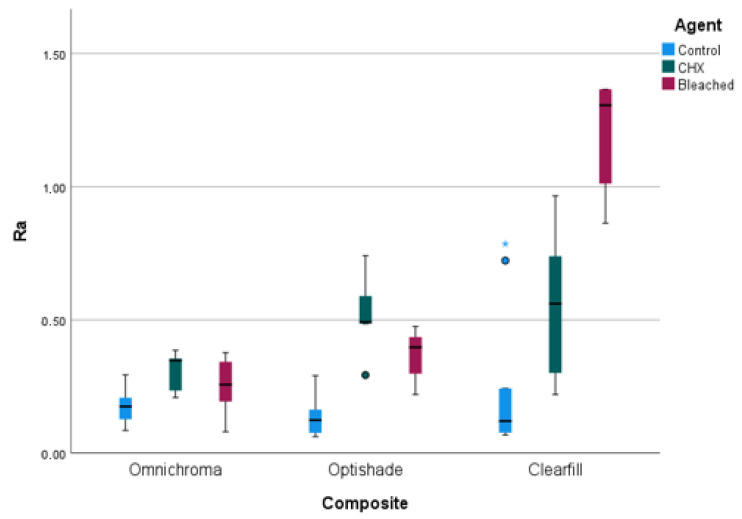
Box chart of the groups. * indicates a statistically significant difference in surface roughness (Ra value) for Clearfil resin composite compared to other materials (*p* < 0.05).

Figure 2 presents the box graph of the Ra distribution of composite resins. According to this distribution, the Clearfill composite resin showed an obvious interfacial roughness value. The Omnichroma composite resin shows the lowest surface roughness values.

In Table 3, optically direct images of the groups are given. In the direct images from the profilometer, the Clearfill composite resin discs showed the highest surface roughness after bleaching. The lowest surface roughness image was obtained for the Optishade composite resin.

## 4. Discussion

In this in vitro study, the surface roughness of a universal single-tone composite resin, a universal group-tone composite resin, and a conventional composite resin was compared after chlorhexidine staining and bleaching of the stained resins. A new-generation polishing rubber with diamond particulate was used as the standard for each group.

Surface roughness is an important factor in dentistry. The disadvantages of surface roughness include the formation of bacterial biofilms, easy discolouration of the surface, loss of aesthetics, and, in advanced cases, fractures and cracks, especially in composites used for the anterior region [19]. Discolouration is an inevitable disadvantage of composite resins, but in order to preserve the initial colour as much as possible, minimal surface roughness is required. A discoloured composite resin may require repair or remodelling. Many different devices are used for surface roughness measurements. Nowadays, profilometer devices that can provide 3D images are popular for these measurements.

In this study, low surface roughness was obtained for all composite groups in the control subgroups compared to the other treated subgroups. In the Omnichroma and Optishade composite resin groups, there was a relative increase in surface roughness after chlorhexidine immersion and bleaching compared to the control groups. In the Clearfill groups, it was found that the surface roughness measurement values after chlorhexidine immersion showed a sudden jump following the bleaching process, providing a much higher surface roughness value.

One of the production purposes of composite resins with nano or supranano contents is to achieve maximum surface smoothness. In the present study, the conventional composite resin showed the highest surface roughness. Based on this, it would be better to use a composite resin with a nano or supranano filler content instead of conventional composite resins in anterior composite resin restorations. The reason for this claim is the importance of the composite content. Today, maximum surface smoothness is obtained due to the minimal particle and maximal filler content of the newly produced composites. It is concluded that the reason why conventional composite resins give high roughness values compared to universal composites is that the filler ratio is lower and the particle ratio is higher [7,8,9,10,11,12,13,14,15,16,17,18,19,20].

In a previous study, the surface roughness values of a Clearfill hybrid composite, a supranano particle composite resin, and a nanohybrid particle composite resin were observed after polishing. Unlike our study, the Clearfill composite resin showed the highest surface roughness value among the composites whose surface roughness was only observed after polishing. The lowest surface roughness value was shown by the supranano particle composite resin [21]. The different values in this study are based on the same grit number as the polishing material used in our study.

There are studies that compared the effect of different bleaching agents on the surface roughness of composite resins. Carbamide peroxide and hydrogen peroxide are the most commonly used agents in studies at different concentrations. In a study in which two different concentrations of of carbamide peroxide and one concentration of hydrogen peroxide were used, it was observed that hydrogen peroxide had a greater effect on surface roughness [22]. Other study that compared the surface roughness values of nanohybrid and microhybrid composite resins after three different bleaching procedures (40% hydrogen peroxide, 35% carbamide peroxide, and 6% hydrogen peroxide) found no significant mean differences between them [23]. Another study that compared a microhybrid composite and a nanohybrid composite treated with two different bleaching agents (16% carbamide peroxide and 40% hydrogen peroxide) showed that the microhybrid composite resin had greater surface roughness than the nanohybrid composite resin. This study also demonstrated that hydrogen peroxide has a greater effect on the surface roughness of composite resins. This may mean, similarly to our study, that hybrid composites show the same roughness values as conventional composite resins [24]. A study that compared the bleaching effects of carbamide peroxide and hydrogen peroxide on a nanofill composite which was immersed in two different solutions similarly showed that hydrogen peroxide had a more significant effect on surface roughness [25]. Our bleaching agent was hydrogen peroxide, and after using it, our results showed that all groups displayed a significant mean surface roughness value after bleaching. Regarding bleaching agents, according to the studies discussed, carbamide peroxide was associated with lower surface roughness values than hydrogen peroxide. The reason for this, as mentioned in the Introduction, is that hydrogen peroxide has a more acidic structure and can dissolve the organic matrix to a greater extent.

Among the many mouthwashes used today, CHX is accepted as the gold standard and is widely used today, buy its discolouring effects on composite resins have been demonstration by many studies [12,26]. However, there are few studies that have indirectly demonstrated the effect of chlorhexidine mouthwash on the surface roughness of composite resins. One of the studies that supports this hypothesis found that, similarly to our study, chlorhexidine mouthwash significantly affects surface roughness, particularly the Clearfill conventional composite resin [27].

Surface roughness may also depend on the duration of direct contact with the mouthwash. In this study, the immersion time of 24 h corresponded to two years of regular mouthwash use. However, different results were obtained in other studies. In one study, it was reported that composite resins exposed to chlorhexidine five times a week for 1 min per day for a total of 3 weeks showed lower surface roughness [28]. In another study, composite resins were kept in chlorhexidine for a total of 30 days with 1 min of exposure every 12 h. In this study, surface roughness was reported to be lower [29]. In our study, we tested exposure in a patient with continuous use, which corresponds to 2 min of exposure per day in accordance with the recommended usage. As a result, it was shown that surface roughness increased significantly in all groups. Patients exposed to chlorhexidine for less time are likely to show less surface roughness.

Another factor that may affect the surface roughness is the finishing and polishing processes. In this article, a single type of polishing process was performed for each group in order to avoid affecting the surface roughness values indirectly. In many studies, it has been reported that diamond-containing Twist Dia finishing and polishing discs provide the maximum surface smoothness. Particularly in universal composite resins, diamond-containing finishing and polishing processes have been shown to give the best results [30,31]. In this article, a Twist Dia finishing and polishing set was used for each group, but no effects were observed.

This study has many limitations. Within the framework of limitations, chlorhexidine and hydrogen peroxide, the bleaching agent, have both been proven to increase surface roughness. This should be verified for other mouthwashes and bleaching agents. The effect of finishing and polishing processes, which is another limitation, can be explored by including them in further studies. Different remediation methods for composite resins affected by mouthwashes and bleaching agents can be added to these studies. For example, re-finishing and polishing can be performed. In addition, the minimal removal of the affected surface and the reconstruction of the affected surface with the appropriate method can also be applied in these studies.

## 5. Conclusions

Considering the limitations of this study, the conclusions we should obtain from this study as clinicians are as follows:

1. In line with the findings obtained as a result of the in vitro study, higher surface roughness values after bleaching are seen in the conventional composite resins, and thus universal composite resins should be preferred, especially in anterior aesthetic areas.
2. Since the surface roughness value after chlorhexidine discolouration is seen to increase for the Optishade composite resin, it should be noted that universal composite resins used in aesthetic areas may need more stringent maintenance, or other composite resins may be preferred.
3. Considering the destruction of composite resin restorations after bleaching, restoration renewal may need to be considered.
4. The use of chlorhexidine mouthwashes should be carefully considered and the duration of use should be determined according to the presence of restoration in the mouth. It may not be as effective with inappropriate use.
5. In order to support this study, experiments with different bleaching agents followed by a combination of CHX mouthwashes with different grain contents and finishing and polishing types are needed.

## Figures and Tables

**Table 1:** Comparison and data analysis of Ra measurements according to composite, CHX, and bleaching methods.

	Control	CHX	Bleached
	Ort. ± S.D	Ort. ± S.D	Ort. ± S.D
Omnichroma	0.1779 ± 0.0655 (0.1751)	0.3063 ± 0.0792 (0.3477)	0.2533 ± 0.0934 (0.257)
Optishade	0.1337 ± 0.072 (0.1238)	0.5204 ± 0.1637 (0.492)	0.3654 ± 0.1046 (0.3971)
Clearfill	0.2442 ± 0.2737 (0.1203)	0.5581 ± 0.2973 (0.5613)	2.569 ± 3.2097 (1.3056)
	Test Statistic/*p*	Test Statistic/*p*	Test Statistic/*p*
Between CHX Groups	4.748 †/0.019 *	23.133 †/<0.001 *	13.610/0.001 *
Between Composite Groups	2.495/0.287 *	2.186 †/0.152 *	12.643/0.002 *

* *p* < 0.05; †: ANOVA.

**Table 2:** Three-dimensional imaging of the different groups.

Omnichroma		
Control	CHX	Bleached
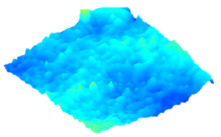	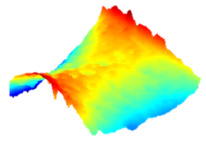	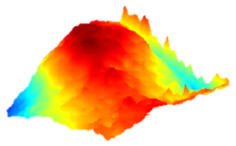
Optishade		
Control	CHX	Bleached
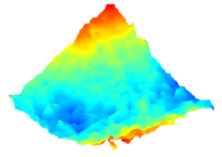	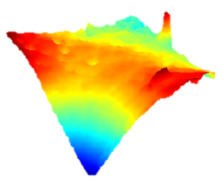	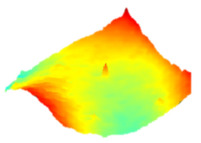
Clearfill		
Control	CHX	Bleached
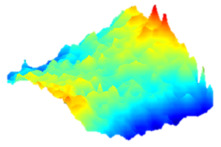	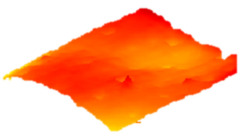	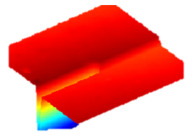
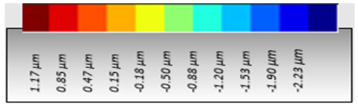		

**Table 3:** Optical images of groups.

Omnichroma		
Control	CHX	Bleached
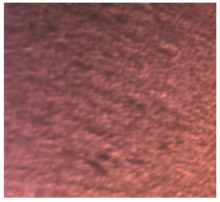	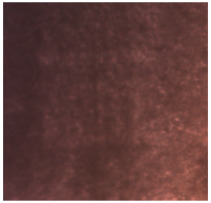	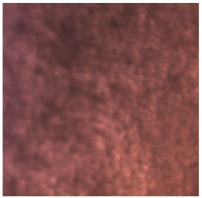
Optishade		
Control	CHX	Bleached
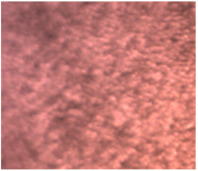	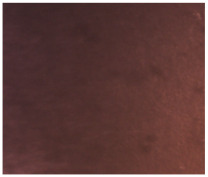	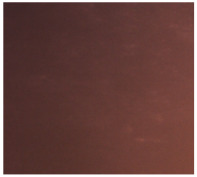
Clearfill		
Control	CHX	Bleached
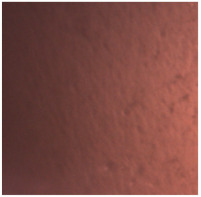	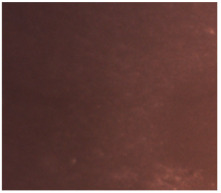	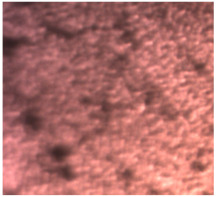

## Data Availability

The data presented in this study are available on request from the corresponding author.
